# Supplementary material for: Understanding the mesoscopic scaling patterns within cities
Source: Sci Rep. 2020 Dec 3;10:21201. doi: 10.1038/s41598-020-78135-2 (PMC7712915; doi:10.1038/s41598-020-78135-2)
Supplement: Supplementary file 1 — Supplementary material 1 [file 41598_2020_78135_MOESM1_ESM.pdf]

Supplementary Information for
Understanding the mesoscopic scaling patterns within cities

Lei Dong,[1,2] Zhou Huang,[1] Jiang Zhang,[3] and Yu Liu[1,*]

[1]*Institute of Remote Sensing and Geographical Information Systems,*
School of Earth and Space Sciences,
Peking University, Beijing 100871, China

[2]*Senseable City Lab, Department of Urban Studies and Planning,*
Massachusetts Institute of Technology, Cambridge, MA 02139, USA

[3]*School of System Science, Beijing Normal University, Beijing 100875, China*

[*] liuyu@urban.pku.edu.cn

SUPPLEMENTARY TABLES

City	N	Center (lat., lon.)	Population (10^4)	Mobile Phone Data Coverage (%)
Beijing	198	39.907, 116.391	2,171	77.2
Shanghai	94	31.231, 121.471	2,415	63.2
Chengdu	96	30.659, 104.064	1,466	74.0
Nanjing	77	32.043, 118.779	823	63.7
Zhengzhou	86	34.747, 113.654	957	75.2
Hangzhou	59	30.242, 120.204	902	72.0
Suzhou	57	31.302, 120.581	1,062	81.7
Jinan	57	36.672, 116.989	700	54.9
Shenzhen	39	22.540, 114.060	1,303	111*
Xi'an	63	34.261, 108.942	871	84.4

Supplementary Table 1 Descriptive statistics of ten cities. N is the number of grid cells used in the analysis. The coordinates of urban center are collected from Wikipedia; population size is derived from the city yearbook. The mobile phone data coverage equals our mobile phone samples divided by the urban population. *: Because Shenzhen has a large number of floating population making the official statistics of the population underestimate the actual size of the population. This is why we find that the number of mobile phone users is higher than the official urban population.

Cell size	$\langle\beta_{infra}\rangle$	$\langle R^2_{infra}\rangle$	$\langle\beta_{firm}\rangle$	$\langle R^2_{firm}\rangle$	$\langle\beta_{POI}\rangle$	$\langle R^2_{POI}\rangle$
1km	0.753	0.745	1.29	0.730	1.25	0.738
1.5km	0.799	0.816	1.26	0.793	1.25	0.783
2.0km	0.833	0.839	1.25	0.822	1.26	0.808
2.5km	0.854	0.891	1.24	0.858	1.27	0.862

Supplementary Table 2 Cell sizes and scaling results. All values are averaged for ten studied cities.

City	Moran's I
Beijing	0.649
Shanghai	0.676
Chengdu	0.716
Nanjing	0.588
Zhengzhou	0.703
Hangzhou	0.556
Suzhou	0.558
Jinan	0.740
Shenzhen	0.344
Xi'an	0.719

Supplementary Table 3 Moran's I. To calculate the Moran's I of the spatial distribution of active population, we use the lctools package (https://cran.r-project.org/web/packages/lctools/index.html) in R.

City	Housing area per capit (m^2)
Beijing	29.26
Tianjin	30.00
Taiyuan	29.00
Hohhot	31.35
Dalian	27.30
Changchun	29.20
Harbin	27.00
Shanghai	30.90
Hangzhou	34.30
Ningbo	25.00
Hefei	28.80
Fuzhou	32.10
Xiamen	33.40
Nanchang	29.04
Zhengzhou	31.00
Wuhan	33.50
Guangzhou	22.50
Shenzhen	27.90
Haikou	29.80
Chengdu	32.90
Guiyang	22.70
Xi'an	33.00
Xining	25.80
National Average	32.91

Supplementary Table 4 Housing area per capita of Chinese cities. Data from [1] and City Statistical Year Book.

Category	Feature
Individual level	# of stay point
	# of unique date of stay point
	weekday # of stay point / weekend # of stay point
	weekday daytime # of stay point / weekday nighttime # of stay point
Cluster level	weekday # of stay point / weekend # of stay point (each cluster)
	weekday daytime # of stay point / weekday nighttime # of stay point (each cluster)
	# of stay point in each cluster / total # of stay point
	weekday # of stay point (each cluster) / total # of stay point
	weekend # of stay point (each cluster) / total # of stay point
	daytime # of stay point (each cluster) / total # of stay point
	nighttime # of stay point (each cluster) / total # of stay point
	# of other clusters to this cluster before 12:00 (transfer matrix)
	# of this cluster to other cluster before 12:00 (transfer matrix)
	# of other clusters to this cluster after 12:00 (transfer matrix)
	# of this cluster to other cluster after 12:00 (transfer matrix)
Regional level	Region ID
POI level	# of residential point of interests (POI)
	# of working point of interests (POI)

Supplementary Table 5 Main features for home and work location classification. We set 9:00-18:00 as daytime, and the remaining period as nighttime; Monday-Friday as weekday, and Saturday and Sunday are weekend. Note that 'transfer matrix' at cluster level means movement between clusters.

SUPPLEMENTARY FIGURES

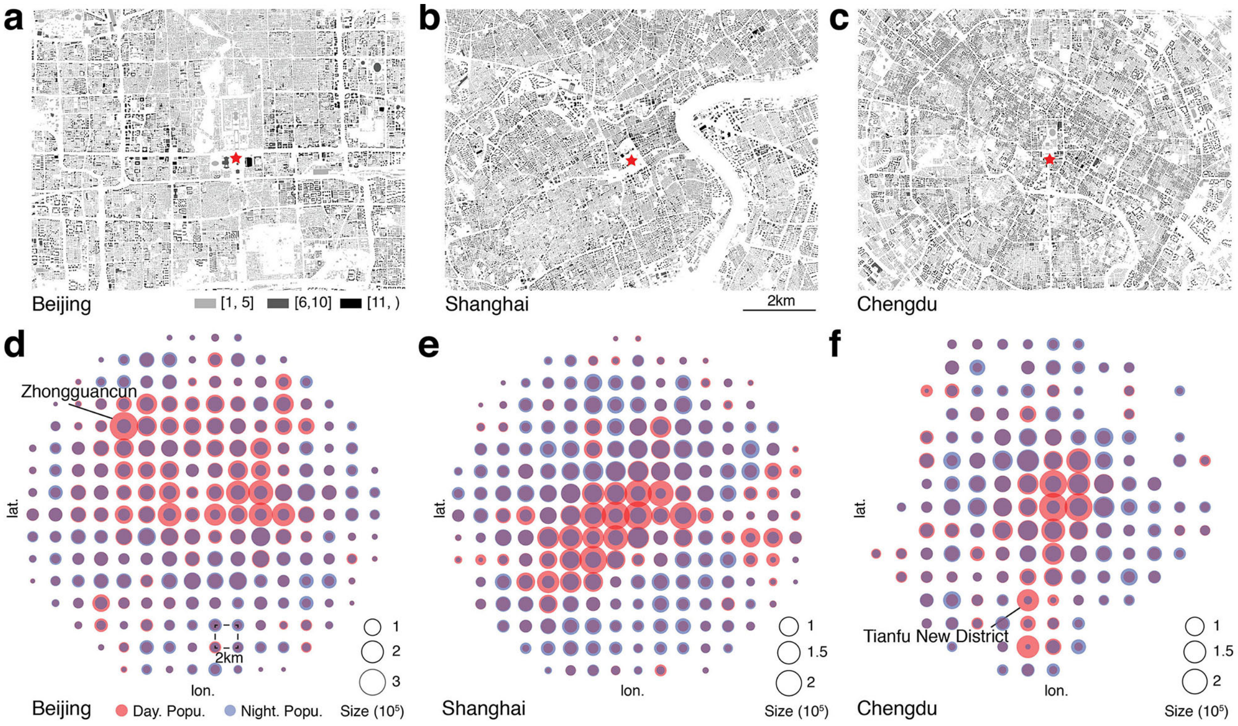

Supplementary Fig. 1 Geographical distributions of buildings and population. **a-c**,Geographical layout of buildings of Beijing (a), Shanghai (b), and Chengdu (c). We classify the floor number into three categories: 1-5, 6-10, and ≥ 11. City centers are marked with a star symbol. These maps were generated by QGIS 2.18 (https://qgis.org/). **d-f**, Daytime and nighttime population distributions of Beijing (d), Shanghai (e), and Chengdu (f). The circle sizes represent the population sizes; the red and blue colors represent the daytime and nighttime populations, respectively. The places where the red circle is larger than the blue circle represent the area where the daytime population is more than the nighttime population, and most of them are job centers.

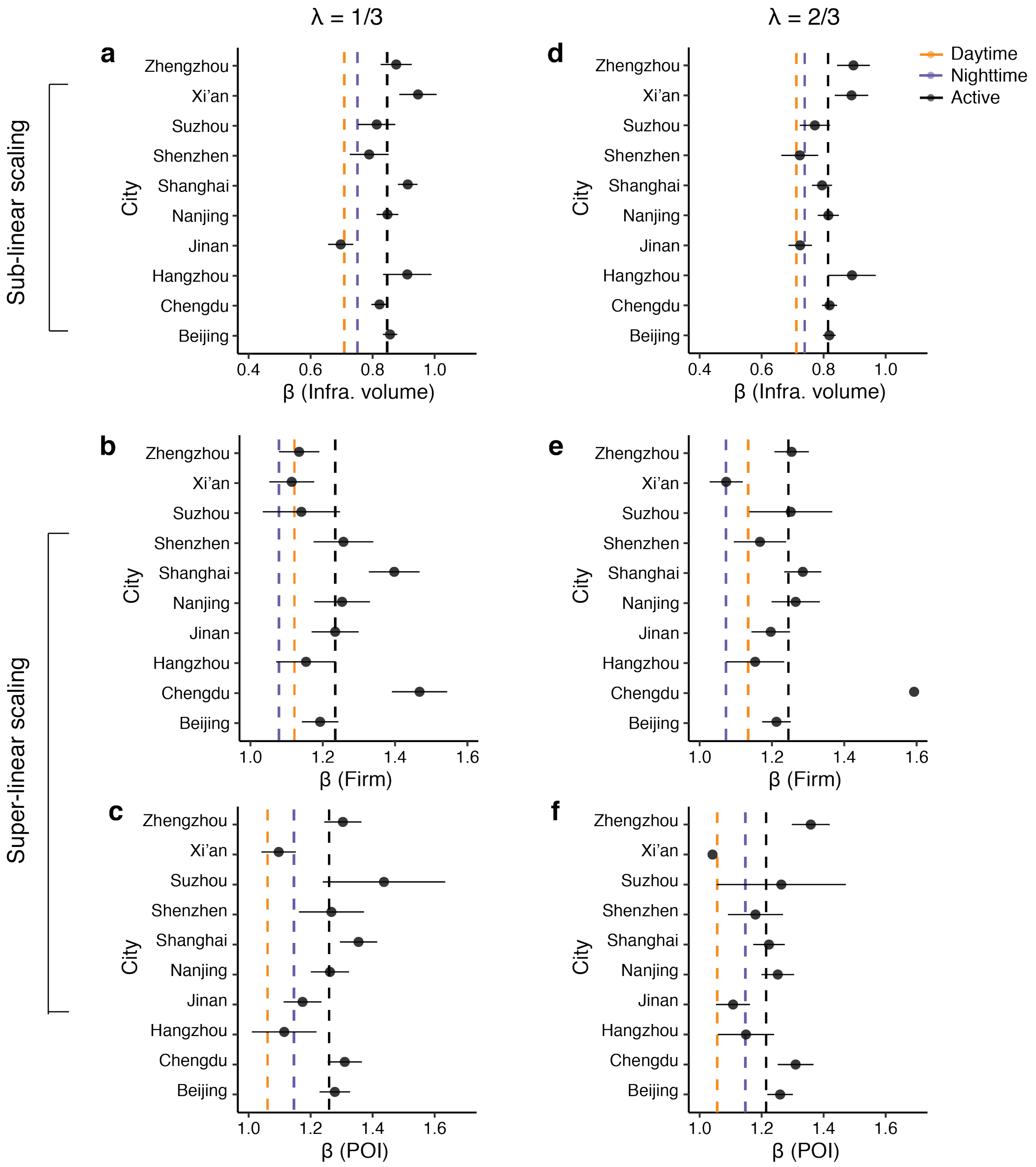

Supplementary Fig. 2 λ **and scaling exponents. a-c**, the average scaling exponent $\beta =$ 0.843, 1.23, and 1.26 for infrastructure (a), firms (b), and POIs (c), respectively ($\lambda = 1/3$); **d-f**, the average scaling exponent $\beta = 0.810$, 1,24, and 1.21 for infrastructure (d), firms (e), and POIs (f), respectively ($\lambda = 2/3$). All these results are similar to the main text ($\lambda = 1/2$).

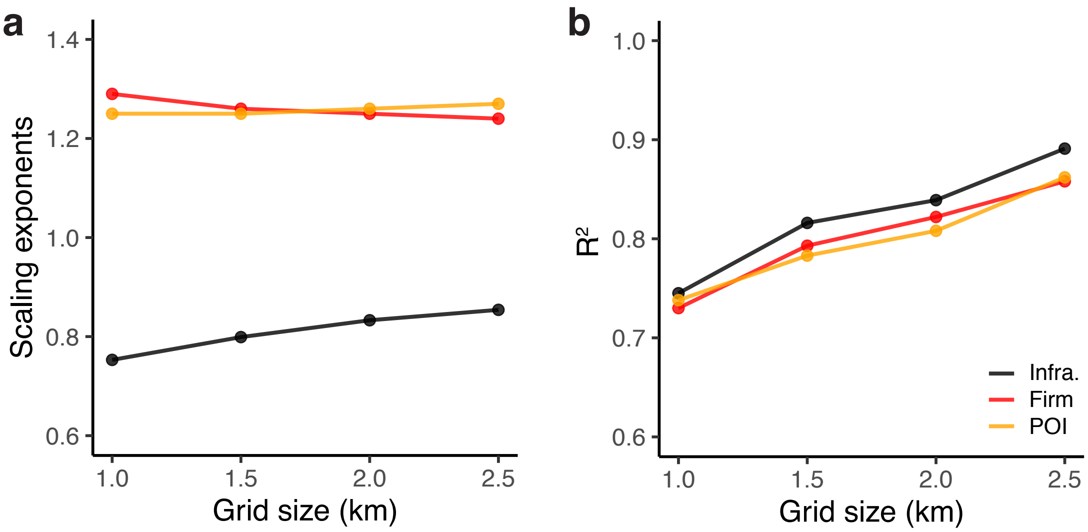

Supplementary Fig. 3 Cell sizes and scaling results. **a,** Scaling coefficients. **b,** R^2s. All values are averaged for ten studied cities. We use 2km grid in the main text.

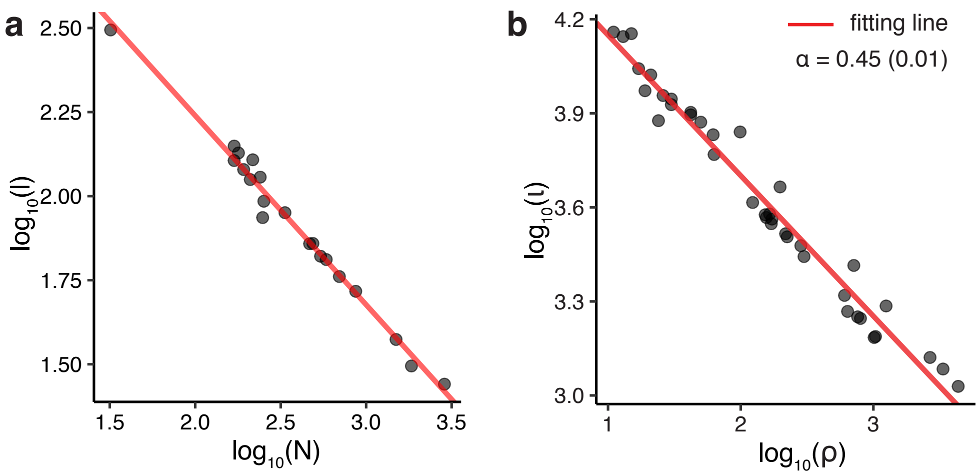

Supplementary Fig. 4 Scaling between population density and average infrastructure length. **a**, Road network data for Eq. (3), detailed in the main text. **b**, Scaling relation $\ell \sim \rho^{-\alpha}$ of simulated data [$\alpha = 0.448(0.013)$, $R^2 = 0.970$]. We generate 20,000 points under a two-dimensional Gaussian distribution within an $L \times L$ space, connect each point to its n ($n = 3$) nearest neighbor, and calculate the relation between point density ρ and average edge length ℓ by $L/10 \times L/10$ grid cells.

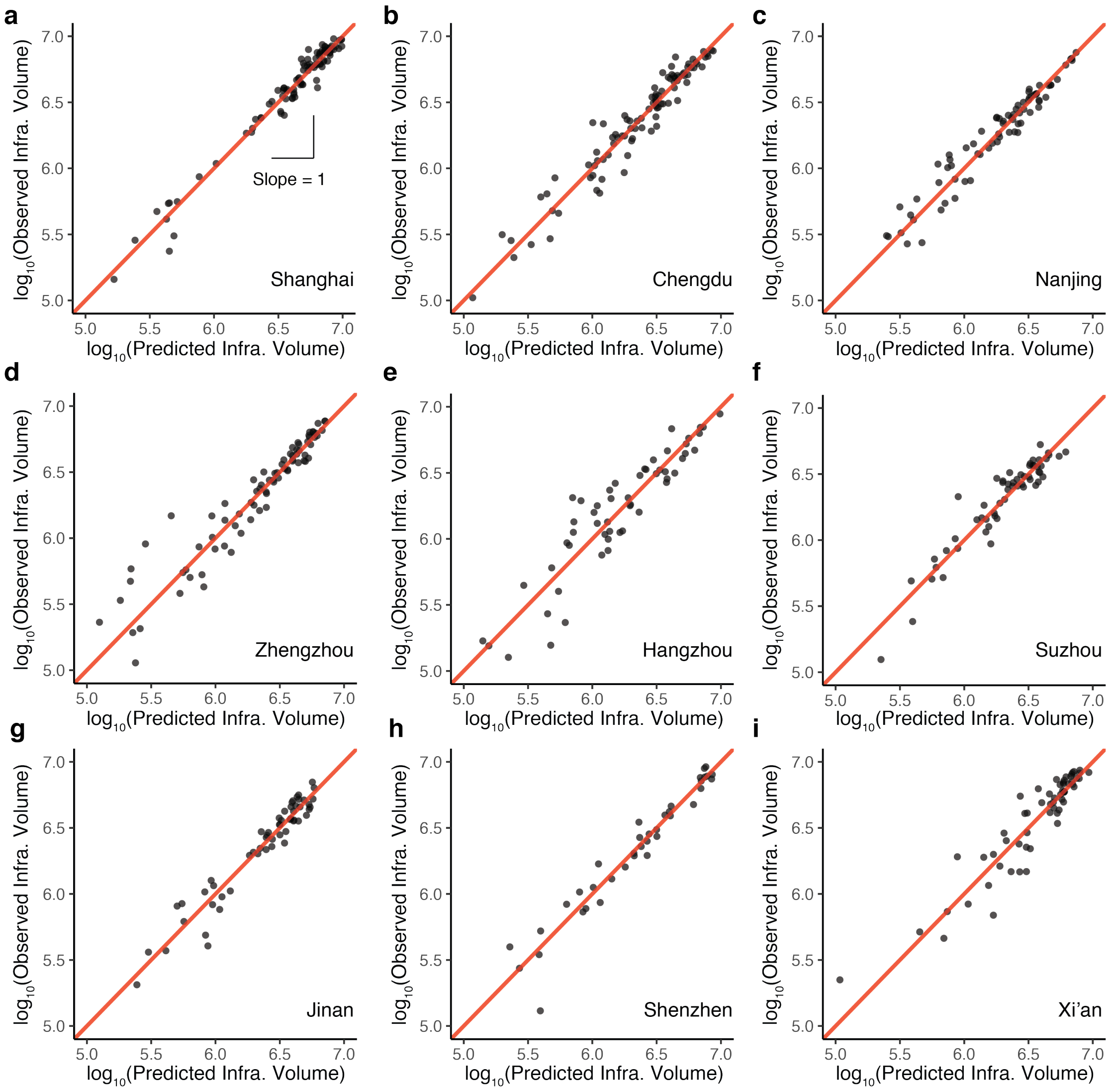

Supplementary Fig. 5 Predicted infrastructure volume and observed infrastructure volume. Similar to Fig. 3c in the main text.

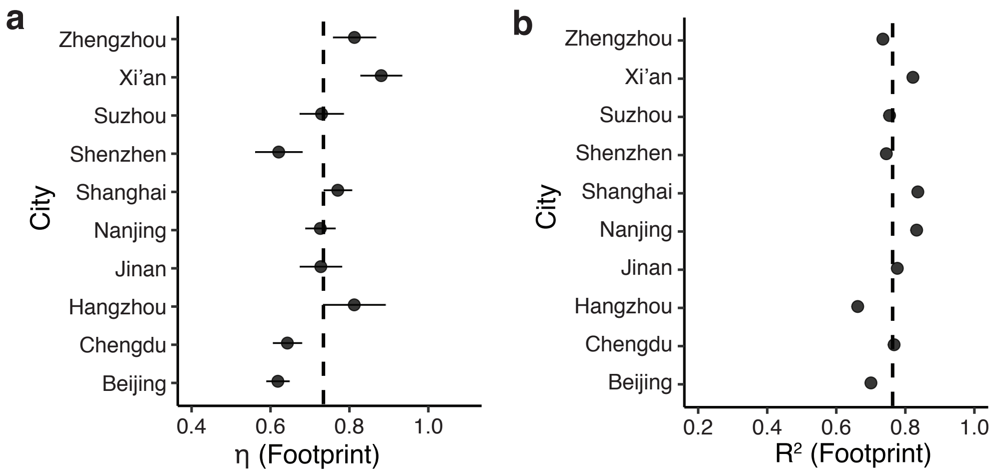

Supplementary Fig. 6 Sub-linear scaling between footprint area A and active population AP. a, β ($\pm$ one standard error). **b**, R^2. The average values are labeled with vertical dashed lines.

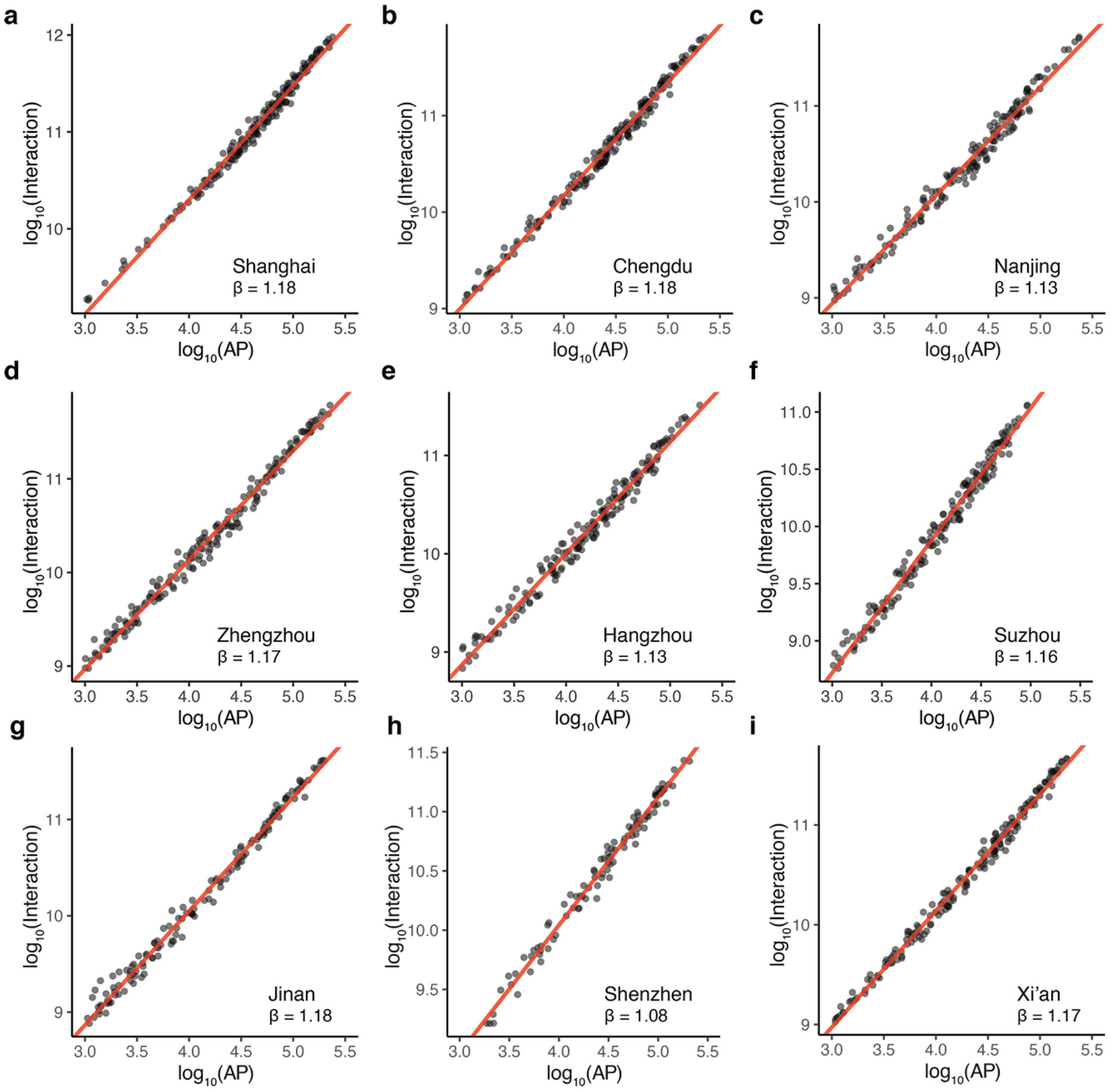

Supplementary Fig. 7 Super-linear scaling predictions. Scatter plot and fitting results between active population size and interactions ($\gamma = 1$ and $k = 1$).

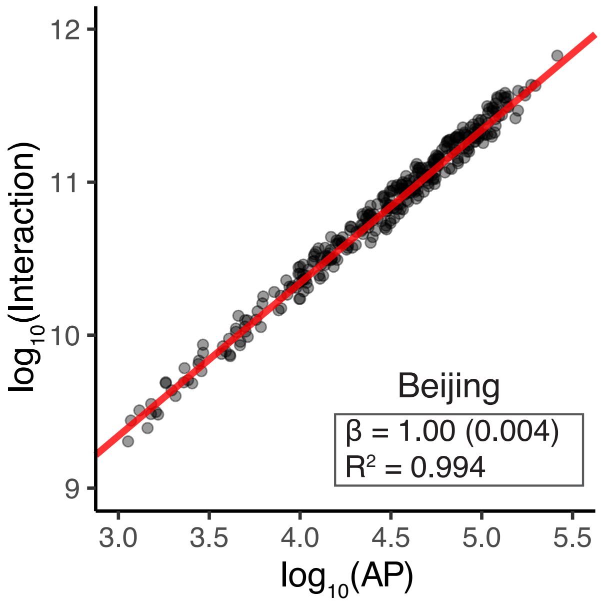

Supplementary Fig. 8 Shuffling population distribution and scaling exponent. Scatter plot and fitting results between active population size and interactions after randomly swapping the values of population in cells ($\gamma = 1$ and $k = 1$). The scaling exponent is 1, indicating a linear relationship between AP and interaction.

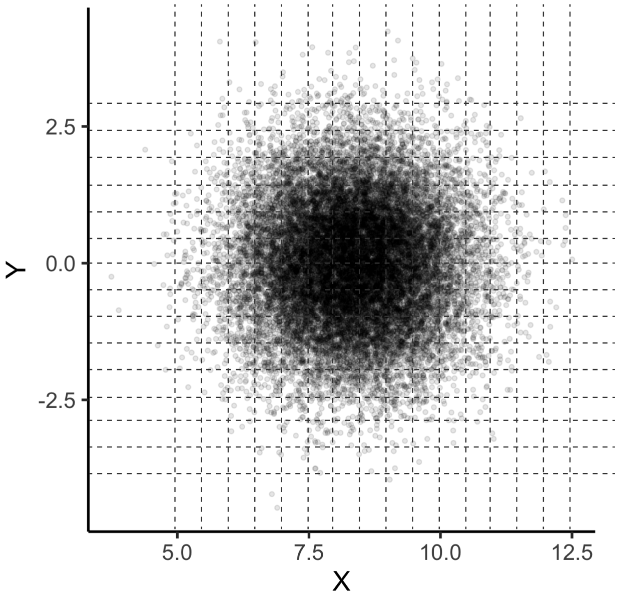

Supplementary Fig. 9 Two-dimensional Gaussian distribution ($\mu = 0$, $\sigma = 1$). We generate 1×10^5 points under a two-dimensional Gaussian distribution with the mean $\mu = 0$ and the standard deviation σ varying from 0.25 to 4. We then partition the space by 0.5×0.5 grid cells and calculate the interaction between each cell pair based on the gravity equation ($\gamma = 1$).

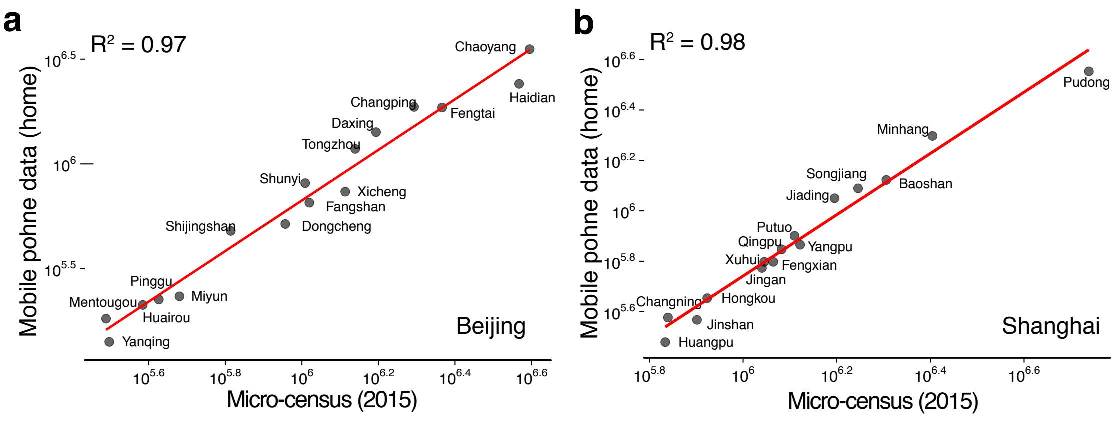

Supplementary Fig. 10 Mobile phone inferred home locations and micro-census. The 1% national population survey (micro-census) was conducted in 2015, the same year of our mobile phone dataset. At the district level, the R^2s of the log-log linear regression are 0.97 for Beijing (a) and 0.98 for Shanghai (b), respectively.

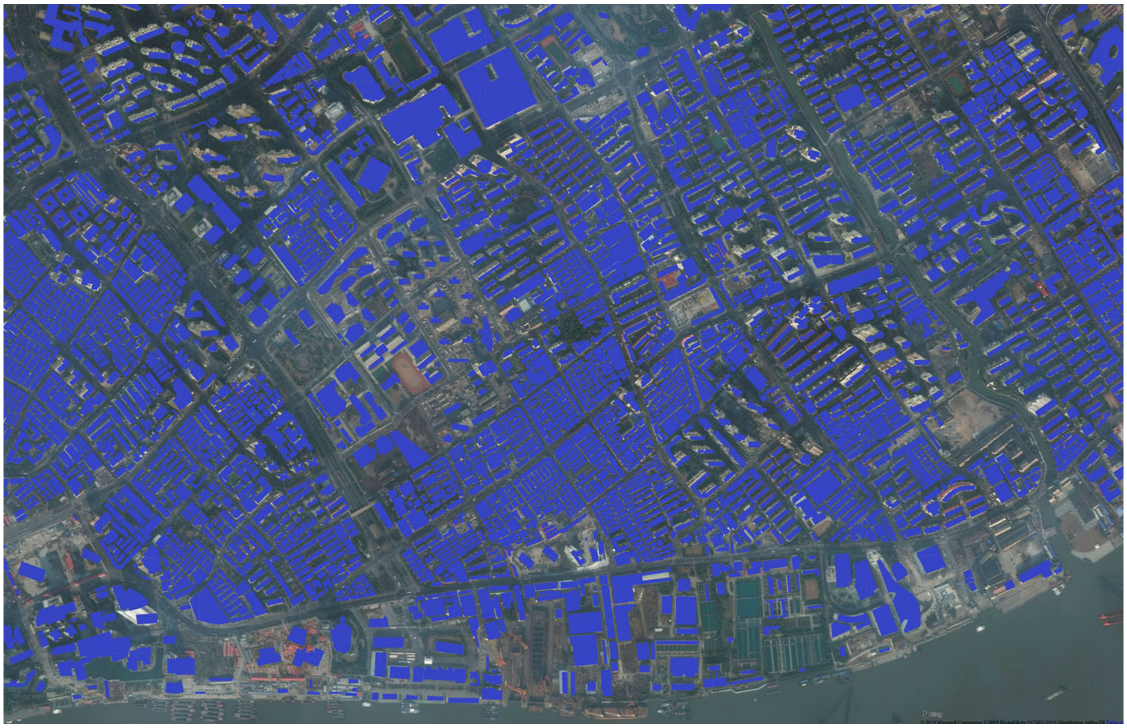

Supplementary Fig. 11 Building footprint with satellite imagery (Shanghai). Satellite image copyright Microsoft (Bing Map, https://www.bing.com/maps/).

---

[1] Yanqun Zhang. Estimation on the relative bubble of the housing price and per capita housing area in 24 big and medium cities of china. *West Forum*, 25(1), 2015.
